# Handwriting performance in the absence of visual control in writer's cramp patients: Initial observations

**DOI:** 10.1186/1471-2377-6-14

**Published:** 2006-04-04

**Authors:** Vihren Chakarov, Sibylla Hummel, Florian Losch, Jürgen Schulte-Mönting, Rumyana Kristeva

**Affiliations:** 1 Neurological Clinic, University Freiburg, Breisacherstraße 64, 79106 Freiburg, Germany; 2 Center of Biomedical Engineering, Bulgarian Academy of Sciences, Sofia, Bulgaria; 3 Universitätsklinikum Benjamin Franklin, Berlin, Germany; 4 Dept. Biometry, University of Freiburg, Germany

## Abstract

**Background:**

The present study was aimed at investigating the writing parameters of writer's cramp patients and control subjects during handwriting of a test sentence in the absence of visual control.

**Methods:**

Eight right-handed patients with writer's cramp and eight healthy volunteers as age-matched control subjects participated in the study. The experimental task consisted in writing a test sentence repeatedly for fifty times on a pressure-sensitive digital board. The subject did not have visual control on his handwriting. The writing performance was stored on a PC and analyzed off-line.

**Results:**

During handwriting all patients developed a typical dystonic limb posture and reported an increase in muscular tension along the experimental session. The patients were significantly slower than the controls, with lower mean vertical pressure of the pen tip on the paper and they could not reach the endmost letter of the sentence in the given time window. No other handwriting parameter differences were found between the two groups.

**Conclusion:**

Our findings indicate that during writing in the absence of visual feedback writer's cramp patients are slower and could not reach the endmost letter of the test sentence, but their level of automatization is not impaired and writer's cramp handwriting parameters are similar to those of the controls except for even lower vertical pressure of the pen tip on the paper, which is probably due to a changed strategy in such experimental conditions.

## Background

There is increasing number of investigations in the field of the handwriting research dealing with different aspects of the handwriting. Some of the investigations are dealing with issues focusing largely on motor control, others on cross-cultural aspects of the handwriting and the neural basis of the handwriting production [1-6].

Writer's cramp represents a form of focal dystonia [7] characterized by co-contraction of agonist and antagonist muscles and recruitment of muscles usually not involved in writing [8]. Patients with simple writer's cramp have impairment only of the handwriting. In patients with dystonic writer's cramp additional manual skills can be affected [9]. During writing the patients adopt highly individualized unusual postures of the fingers and the wrist and exert considerable forces in holding and pressing the pen on the paper [9].

Often writer's cramp is present in people who need handwriting to fulfill their professional duties. Typically this spasm increases with the beginning of the handwriting and in some cases can make the continuation of this task impossible after a few words. The often painful muscle spasm can start even immediately after taking the pen [10]. The prolonged disturbance of this important everyday ability can cause considerable psychological problems; some patients need to change their occupation or go on disability.

Handwriting is a complex-skill task. The generation of a single up- or down stroke is associated with a smooth and single-peaked velocity profile and a smooth one-acceleration and one-deceleration profile [5]. These profile characteristics are highly similar for repetitive handwriting movements, which are called automated and are believed to be pre-programmed before the actual execution of the movement [10]. Velocity profiles with higher number of inversions (multi-peaked) can be due to a disturbance in the execution or reflect control during the execution of the movement [10].

Besides the obvious graphological disturbances in patients with writer's cramp, the kinematical analysis of their handwriting also shows differences in comparison with control subjects. Handwriting examination in writer's cramp patients reveals that they have higher vertical pressure, lower writing velocity, shorter stroke frequency, disturbed automated movements even within a single up- or down stroke generation with velocity profiles of the pen movements on the paper with many irregularities and higher number of inversions in velocity (NIV)[10]. Applying low-frequency repeated transcranial magnetic stimulation (rTMS) to reduce the cortical excitability Siebner et al. (1999) [11] obtained a beneficial effect on handwriting parameters in writer's cramp patients expressed in a positive correlation between the decrease in mean vertical writing pressure and improved automation of handwriting movements. They suggested that higher mean vertical pressure in writer's cramp patients is a compensatory reaction and its decrease after rTMS reflects an improvement in force control during writing.

Usually in everyday life handwriting is performed with visual and proprioceptive control. Marquardt et al. (1996) [10] demonstrated that visual control is not required to produce automated handwriting movements and that conscious attention to visual feedback is hampering the elicitation of automated movements. In their study they presented evidence that even in normal subjects the handwriting can be easily perturbed when attention is directed to visual feedback. They suggested that writer's cramp patients pay too much attention towards the handwriting, which can play a role in the pathophysiology of writer's cramp. Therefore, it was of interest to investigate the effects of lack of visual feedback on writer's cramp patients.

The present study investigated the differences between a group of patients with writer's cramp and control subjects during handwriting of a test sentence in the absence of visual control. The handwriting performance data from the study of Kristeva et al. (2005) [12] were used for this purpose.

## Methods

### Subjects

Eight right-handed patients with writer's cramp participated in the study (two female and six male; mean age 42.2 ± 11.7 years). The mean duration of the symptoms was 7.7 ± 5.96 years. The type of dystonia was with finger flexion in six of the patients (in one of them combined with mild wrist flexion and in one with DI-DIII flexion), one with DII extension and one with DI-DII extension. Four patients were with mild pathological symptoms, two with mild to moderate and two with moderate. Six of the patients had simple writer's cramp. Two of the patients had dystonic writer's cramp according to the definition of [7], *i.e*. they had symptoms not only during writing but also during other motor activities (one when cutting with a knife and one when putting on make-up). None of the patients had been treated with botulinum toxin or any other drug 6 months prior to the study. Clinical details about the writer's cramp patients are reported in Kristeva et al. (2005) [12].

Eight healthy right-handed volunteers (two female and 6 males; mean age 41.5 ± 10.9 years) acted as age-matched control subjects. The neurological examinations of the control subjects were normal.

All subjects participated according to the declaration of Helsinki, with informed consent and the approval of the local ethics committee (Nr. 223/97).

### Experimental paradigm

Prior to the experimental session, the patients were interviewed and video-recorded during writing of the sentence used in standard investigations of handwriting "Die Wellen schlagen hoch" ("The waves rise up high") [4,8].

During the experimental session, the individual was sitting in an electrically shielded, dimly lit room. The dominant hand and arm were supported in a rigid cast.

The experimental task was a modification of the standard handwriting examination [9] and consisted in writing the sentence "Die Wellen schlagen hoch" repeatedly for fifty times on a digital board. The intertrial interval varied between 10 and 15 sec – this time was enough for the patient to start the next trial from a complete relaxation.

To minimize eye movement artefacts in simultaneously recorded EEG the individual had to fix his gaze on a green light diode situated in front of him at the eye level. Thus the subject did not have visual control on his handwriting. The absence of eye movements was monitored by vertical and horizontal EOG (*cf*. [12])

Each subject was given several practice trials prior to the experiment until becoming familiar with the electronic pen and reaching the required writing ability on the graphic tablet in the given time window.

The occurrence of cramps during the task was assessed by the experimenter and by subjective report of the patients and controls after the experiment.

### Recordings

The writing performance was recorded using digitising board (WACOM UD-1212) [12]. The graphic tablet was fixed to the right armrest of the ergonomic chair and adjusted individually for the maximal comfort of each subject. The position of the writing pen was detected with a sampling frequency of 166 Hz and accuracy 0.2 mm in both X and Y directions. Data were provided only with the pen lifted above the tablet for less than 12.7 mm, while the pressure sensitivity of the digital board give the z-direction information. The maximum recording time was set at 9 s. The software package CSWin 1.0 for calculation of velocity and acceleration used non-parametric regression methods: Kernel estimators that assure extremely small and negligible distortion of the signals (for more details *cf*. [12]). The calculation of the statistical parameters was based on subsequent segmentation of up and down strokes. The minimal stroke length (1 mm) and the minimal stroke time (50 msec) defined in the segmentation prevented from segmenting slow movements or tremor in many sub-segments.

### Writing performance analysis

The writing performance of each individual was estimated by quantifying the following parameters: number of written characters in the test sentence, duration of the handwriting of the test sentence and duration of the analysis window, number of the pen touches on paper, mean time and trajectory of the pen on paper and of the pen lifted (similar to the in air phenomenon of [6]), absolute velocity of the pen on paper and of the pen lifted, mean vertical pressure. In addition, the quality and the level of automation of the handwriting (single stroke analysis – based on handwriting trace decomposition of one stroke-segments) was investigated: mean stroke frequency and length, mean time period of segment, number of velocity and acceleration segments, number of inversions (NI) in velocity and acceleration and percent of segments with number of inversions in velocity (NIV) equal to 1.

### Statistical analysis o f the writing performance

Statistical significance of the group differences in the dependent variables (set at p < 0.05) based on the mean subject's values for each writing parameter for all performances was assessed by means of T-test. For more detailed statistical analysis and to follow the dynamics of the handwriting related with the effect of the practice during the experimental condition, the data obtained for each parameter in the fifty sentences were divided on nine groups, taking each fifth value and for verification – taking each mean of the five values. In this case a repeated measurement ANOVA was applied with between group factor "group" with two levels (controls and patients) and within group factor "task" with nine levels (each fifth value or each mean of five values in the sequence of 50 data for each handwriting parameter). The between group differences in the slope as well as the correlation of the same data sets were checked.

## Results

Regarding the writing of the test sequence the patients were significantly slower than the controls: the mean time of writing (till the end of the sentence or in case the sentence was not completed – till the end of the 9 sec time window) for patients was 8740.75 ± 90.44 ms and for controls 7728.38 ± 62.29 ms (T-test, p < 0.0024). Only one out of the 8 patients was able to complete the sentence in the given 9 second time window. One of the control subjects was not able to finish the sentence in the given time window although there was no neurological reason for this. The mean number of written characters in the test sentence (from maximal 24 including the intervals between words) was 19.16 ± 2.77 for the patients and 23.12 ± 2.04 for the controls. This difference was significant (T-test, p < 0.0037). To make possible the comparison between patients and controls we investigated only that part of the sentence "Die Wellen schlagen hoch" performed by all of them ("Die Wellen schlage").

**Figure 1:**
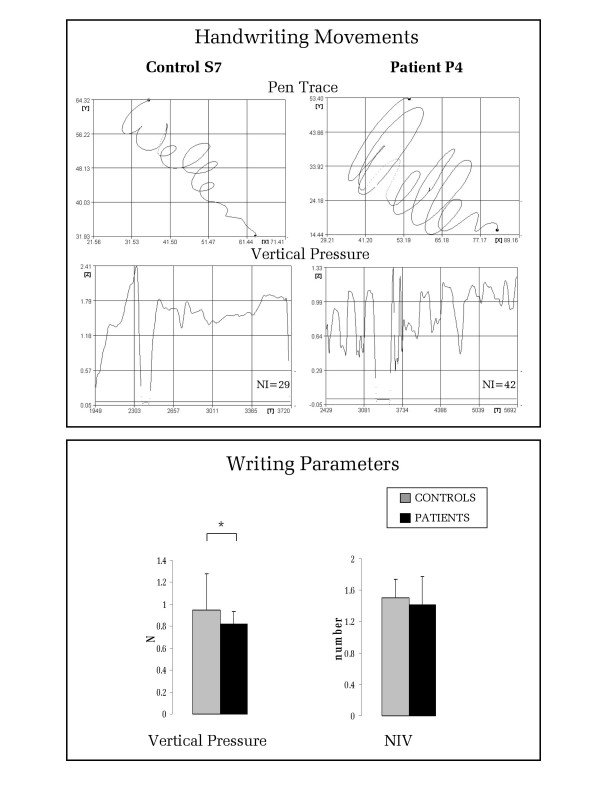
**Original handwriting movements and writing parameters (vertical pressure and NIV)**. *Upper panel*: original data of handwriting movement during writing of the highly automated word "Wellen" from the sentence "Die Wellen schlagen hoch" for control subject S7 (left part) and writer's cramp patient P4 (right part). The upper plots represent the X/Y coordinate plot of the tip of the writing pen on the digitizing tablet. The lower plots are the respective time courses on Z-axis for the vertical pressure. Under each the number of inversions of vertical pressure is given (NI). The continuous line denotes the pen trace on the tablet and the dotted one – the pen trace lifted. Note in the pen trace that the patient has a longer stroke length and trajectory on the paper with longer pen lift. Note also that the time course pattern of the vertical pressure for the patient is characterized by larger number of segments with more NI and lower vertical pressure. *Lower panel*: mean group values and standard deviations (error bars) for 7 controls (grey column) and 7 patients (black column) for vertical pressure and number of inversions in velocity per stroke (NIV). The asterisk denotes a statistically significant difference between both groups (p < 0.05). Note the statistically significant lower vertical pressure for the patients.

Fig. 1 (upper panel) shows the original data of the pen trace and the vertical pressure time course profile for one control subject (S7) and one patient (P4) during writing of the highly automated word "Wellen". It can be seen that the patient has a tendency for longer trajectory of the pen trace, longer movement duration and lower vertical pressure with a complex pattern and with a higher number of vertical pressure inversions (NI).

When the results were pooled for all patients and controls the patients showed significantly lower mean vertical pressure (T-test, p < 0.026, *c. f*. Table 1 and Fig 1 lower panel). Further, the number of inversion in velocity (NIV) per stroke was not significantly different between the two groups (*cf*. Fig. 1, lower panel).

The two-way ANOVA analysis of each fifth value and for each mean of the five values in the 50 realizations set did not reveal any significant effect of both factors and interaction between them. No significant between group differences were found in the analysis of the slope and regression of these data.

## Discussion

The present study investigated the handwriting parameter differences between a group of patients with writer's cramp and control subjects during handwriting of a test sentence in the absence of visual control. It shows that the writer's cramp patient group was significantly slower than the control one and that the mean vertical pressure of the pen tip on the paper was lower. The mean time of writing till the end of the sentence for patients was longer than for controls (T-test, p < 0.0024). During writing in the absence of visual control the patients exert significantly lower mean vertical pressure than controls (T-test, p < 0.026). No other significant handwriting parameter differences were found.

Handwriting is a skilled complex movement in which strokes are the fundamental units. During this activity the brain motor control system has to maintain simultaneously three main components in adequate proportions: static component (gripping and holding the pen), horizontal component (producing up and down strokes) and vertical component (pressing the tip of the pen on the paper) [11]. The kinematical analysis reveals that the generation of a single up or down stroke is characterized with smooth single-peaked velocity and acceleration profile with one acceleration and one deceleration phase [9]. These are the defining characteristics of *automated *movement, which are conceptualized as being pre-programmed before the actual execution of the movement (open-loop control) and with normal frequency ranges between 4 and 6 Hz. In contrast, multi-peak velocity profiles with high NIV can either indicate a disturbance of the movement or reflect control during execution [11].

In our experimental setting, when considering the execution of the entire test sequence, the patients were significantly slower than the controls (T-test, p < 0.0024) and they could not reach the endmost sign of the test sentence, as the controls did (T-test, p < 0.002).

Surprisingly, the vertical writing pressure of our patients was significantly lower than that of the controls. Furthermore, the NIV did not show any statistically significant differences between patients and controls. At first sight these results are in contradiction to the results of Siebner et al. (1999) [11] who showed significantly higher vertical writing pressure in their patients compared to the controls. These authors also showed a significantly higher NIV in the patients suggesting decreased automatization. These apparent discrepancies can be resolved if one takes into consideration the instruction given to our subjects: to write the sentence with eyes fixed on a point in front of them, *i.e*. without a visual feedback. Normally the writing control represents a proprioceptive and visual feedback control system. It is possible that in the absence of visual feedback the gain for proprioceptive feedback from the periphery is increased, leading to different sensorimotor integration for both patients and controls. However, the patients show again an abnormal sensorimotor integration as compared to the controls, *i.e*. they have a wrong feedback update. The sensorimotor functions altered are the vertical pressure, which is lower in comparison to the controls (p < 0.026) and the time of handwriting till the end of the test sentence, which is longer than in the controls (p < 0.002). It is of interest that in the absence of visual feedback the number of inversions of velocity per stroke is not significantly different than that in normal subjects, suggesting that the level of automatization in the absence of visual feedback is normal. Thus our findings provide new experimental evidence for impaired sensorimotor integration in the absence of visual feedback in writer's cramp patients.

Another contributing factor may be the attention. Handwriting is not a basic movement pattern, but a learned skill with high impact of motor strategies, whereby attention processes are crucial in its organization [9-11]. These authors found that in writer's cramp patients a lower attention to the details of handwriting modulates the degree of disturbance. This effect is explicitly used in their handwriting approach. These authors even believe that the interference of a movement problem and the elevated attention to the consequences lead to disturbances like writer's cramp.

It is possible that during standard writing parameters examination as used in the study of Siebner et al. (1999) [11] the dystonic patients focus too much attention on their writing and use a higher vertical pressure. Therefore the higher vertical pressure can be considered rather as a compensatory strategy than as a primary damage. In the absence of visual feedback the phenomenon is reverse and patients do not use the same strategy. The finding that in the absence of visual feedback patients have lower vertical pressure but still have excessive muscular activity confirms the suggestion of Siebner et al. (1999) that the vertical pressure is rather a compensatory strategy[11].

As observed in the clinical practice and also by Mai and Marquardt (1994) patients with writer's cramp exert considerable forces in holding and pressing the pen on the paper [9]. Furthermore, in writer's cramp patients grip force control is impaired [15,16], although more recent studies have refuted some of these findings [17]. Therefore, it will be of interest in future studies to understand the relationship between pen grip force and vertical pressure. This requires synchronization of the pen grip force trace and the handwriting trace. It will be of interest in future studies to investigate a larger sample size. Furthermore, it is necessary in further study to have as well a control condition: writing with visual feedback.

## Conclusion

Taken together, our findings indicate, that during writing in the absence of visual feedback, writer's cramp patients are slower and can not reach the endmost letter of the test sentence. The level of automatization is not impaired and writer's cramp handwriting parameters are similar to those of the control group except the lower vertical pressure of the pen tip on the paper, which is probably due to a changed strategy in such experimental conditions.

## Abbreviations

NI – number of inversions

NIV – number of inversions in velocity

rTMS – repetitive transcranial magnetic stimulation

## Authors' contributions

VC carried out the handwriting analysis studies, participated in the collecting of the experimental data and in the writing the manuscript. SH and FL established the medical diagnoses and collected the experimental data and worked on the MS. JS-M participated in the design of the study and performed the statistical analysis. RK conceived the study and participated in all the steps of the realization of the study. All authors read and approved the final manuscript.

## Pre-publication history

The pre-publication history for this paper can be accessed here:



## Figures and Tables

**Table 1:** Mean Group Values of handwriting parameters

Handwriting Parameter	Controls Mean ± SD	Patients Mean ± SD	T-Test p <
Number of written characters in the test sentence	23.12 ± 2.04	19.16 ± 2.77	0.0037 **
Handwriting duration of the test sentence	7728.38 ± 62.29	8740.75 ± 90.44	0.0024 **
Mean vertical pressure	0.87 ± 0.38	0.78 ± 0.16	0.026 *
Mean stroke frequency	3.96 ± 0.74	3.48 ± 0.70	n.s.
Mean time period of segment	258.2 ± 48.37	291.77 ± 52.39	n.s.
Movement duration	5.08 ± 1.13	6.03 ± 1.15	n.s.
Number of Velocity segments	39.49 ± 8.49	41.72 ± 11.49	n.s.
Number of acceleration segments	30.30 ± 10.97	34.54 ± 12.51	n.s.
Number of inversions in velocity	1.41 ± 0.27	1.37 ± 0.36	n.s.
Number of inversion in acceleration	2.15 ± 0.57	2.31 ± 0.77	n.s.
Mean stroke length	5.82 ± 2.81	7.98 ± 3.14	n.s.
Number of pen touches on paper	16.87 ± 8.54	19.03 ± 10.40	n.s.
Mean time pen on paper	6115.27 ± 979.43	6774.18 ± 1041.05	n.s.
Mean time pen lifted	1518.13 ± 542.47	1735.76 ± 972.12	n.s.
Mean trajectory pen on paper	388.38 ± 169.25	483.51 ± 197.38	n.s.
Mean trajectory pen lifted	95.36 ± 61.81	101.29 ± 57.31	n.s.
Absolute velocity pen on paper	67.40 ± 39.13	74.55 ± 35.48	n.s.
Absolute velocity pen lifted	61.48 ± 23.87	59.82 ± 20.78	n.s.
Percent of segments with NIV = 1	82.63 ± 10.51	84.98 ± 12.89	n.s.

## References

[B1] Van Galen GP, Smyth MM, Muelenbroek RGJ, Hylkema H, Plamondon R, Suen CY, Simner ML (1989). The role of short term memory and the motor buffer in handwriting under visual and non-visual guidance. Computer recognition and human production of handwriting.

[B2] Kao HSR, Ping-Wah H, Robinson L, Yen N-S (1989). Psychophysiological changes associated with chinese calligraphy. Computer recognition and human production of handwriting.

[B3] Meulenbroek RGJ, Van Galen GP, Cooley AM, Beech JR (1988). The acquisition of skilled handwriting: Discontinuous trends in kinematic variables. Cognition and action in skilled behavior.

[B4] Meulenbroek RGJ, Van Galen GP (1990). Perceptual-motor complexity of printed and cursive letters. Journal of Experimental Education.

[B5] Zesiger P, Mounoud P, Hauert CA (1993). Effects of lexicality and trigram frequency on handwriting production in children and adults. Acta Psychologica.

[B6] Rosenblum S, Parush S, Weiss P (2003). The In Air phenomenon: Temporal and spatial correlates of the handwriting process. Perceptual and Motor Skills.

[B7] Marsden CD, Sheehy MP (1990). Writer's cramp. [Review] [37 refs]. Trends Neurosci.

[B8] Berardelli A, Rothwell JC, Hallett M, Thompson PD, Manfredi M, Marsden CD (1998). The pathophysiology of primary dystonia. Brain, BRAIN.

[B9] Mai N, Marquardt C (1994). Treatment of writer's cramp. Kinematic measures as an assessment tool for planning and evaluating training procedures. Advances in handwriting and drawing.

[B10] Marquardt C, Gentz W, Mai N (1996). On the role of vision in skilled handwriting. Handwriting and Drawing Research.

[B11] Siebner HR, Tormos JM, Ceballos-Baumann AO, Auer C, Catala MD, Conrad B (1999). Low-frequency repetitive transcranial magnetic stimulation of the motor cortex in writer's cramp. Neurology, NEUROLOGY.

[B12] Kristeva R, Chakarov V, Losh F, Hummel S, Popa T, Schulte-Moenting J (2005). Electroencephalographic spectral power in writer's cramp patients: Evidence for motor cortex malfunctioning during the cramp. Neuroimage.

[B13] Marquardt C, Mai N (1994). A computational procedure for movement analysis in handwriting. J Neurosci Methods, J.

[B14] Marquardt C, Gentz W, Mai N (1999). Visual control of automated handwriting movements. Exp Brain Res.

[B15] Serrien DJ, Burgunder JM, Wiesendanger M (2000). Disturbed sensorimotor processing during control of precision grip in patients with writer's cramp. Mov Disord, MOV.

[B16] Odergren T, Iwasaki N, Borg J, Forssberg H (1996). Impaired sensory-motor integration during grasping in writer's cramp. Brain, BRAIN.

[B17] Schenk T, Mai N (2001). Is writer's cramp caused by a deficit of sensorimotor integration?. Exp Brain Res.

